# Comparing amorphous silica, short-range-ordered silicates and silicic acid species by FTIR

**DOI:** 10.1038/s41598-022-15882-4

**Published:** 2022-07-09

**Authors:** Ruth Ellerbrock, Mathias Stein, Jörg Schaller

**Affiliations:** Leibniz Centre for Agricultural Landscape Research (ZALF), 15374 Müncheberg, Germany

**Keywords:** Biogeochemistry, Environmental sciences

## Abstract

There is increased interest in the terrestrial silicon cycle in the last decades as its different compounds and species have large implications for ecosystem performance in terms of soil nutrient and water availability, ecosystem productivity as well as ecological aspects such as plant–microbe and plant-animal feedbacks. The currently existing analytical methods are limited. Fourier-transform infrared spectroscopy (FTIR) analysis is suggested being a promising tool to differentiate between the different Si species. We report here on the differentiation of varying Si-species/Si-binding (in synthetic material) using FTIR-analyses. Therefore, we collected FTIR-spectra of five different amorphous silica, Ca-silicate, sodium silicate (all particulate), a water-soluble fraction of amorphous silica and soil affected by volcanic activity and compared their spectra with existing data. A decrease of the internal order of the materials analyzed was indicated by peak broadening of the Si–O–Si absorption band. Peak shifts at this absorption band were induced by larger ions incorporated in the Si–O–Si network. Additionally, short-range ordered aluminosilicates (SROAS) have specific IR absorption bands such as the Si–O–Al band. Hence, SROAS and Si phases containing other ions can be distinguished from pure amorphous Si species using FTIR-analyses.

## Introduction

The terrestrial silicon (Si) cycle is highly important for ecosystem functioning, because Si is a beneficial element for plant growth^1^. Despite the Earth crust consists of silicate minerals, only a small amount of Si is bio-available or reactive. The ultimate source of bio-available or reactive Si on a geological time scale is mineral weathering. These bio-available or reactive Si in soils may be immobilized by adsorption and complexation processes, leached as a function of rainfall and irrigation, and may be incorporated into living organisms^2–4^. The release of different silicon species from dead organic matter established a cycle that controls reactive Si in soils on shorter time scales^5–7^. This is because such amorphous silica is much more soluble compared to silicate minerals^8^. The different Si species in soils are interfering with main soil functions like phosphorus availability^9, 10^ or soil water relations^11, 12^.

Other sources for reactive Si in soils are short-range ordered aluminosilicates (SROAS; e.g. allophanes and imogolites) or short-range ordered silica and amorphous Al:Si phases with different ratios^13^. The currently used methods to analyze different Si phases in soil from different single extraction methods to sequential extraction methods are not very selective but there might be some promising recently developed methods with higher selectivity for single phases^4, 14, 15^. The same holds true for dissolved Si species. The molybdate method measures preferentially monomer and dimer forming a yellow colored β-silicomolybdate complex^16^, but is not that sensitive for higher polymerized species^17^, while the analysis of a liquid phase by e.g. ICP-OES measures the total Si in solution. From the difference between ICP-OES and molybdate method the concentration of polysilicic acid might be estimated roughly. However, a more detailed analysis of both soil and liquid phases would be helpful for interpretation (concentration of different liquid species or differences in quality of e.g. amorphous silica) of several processes in environmental matrices.

Fourier-transform infrared spectroscopy (FTIR) –already used e.g. by Farmer et al.^18^ to characterize the chemical structure of synthetic aluminoosilicate gels- was recently suggested to be a potential tool to analyze certain Si species^19, 20^. For example, compressive stress in the subsurface regions of glasses through exchange of network modifier ions with larger ions induces a red-shift of the Si–O–Si bridging oxygen (BO) stretch band in the IR spectrum (Ref.^20–22^ in Kaya et al.^21^). Furthermore, molecular vibrations induced by IR radiation are a function of binding strength and the mass of the atoms involved. Hence, the absorption bands shifted towards lower wave numbers (WN) as the mass of the atoms increases^18^. Additionally, a decrease in internal order as found for iron oxides results in a broadening of absorption bands^22^. Hence, Si oxide phases containing other ions may be separated from pure silicon bonds as Si–O–Si and Si–OH. However, there is no study comparing different Si species such as different amorphous silica, quartz sand, silica gel, sodium silicate, calcium silicate, SROAS or amorphous Al:Si-phases, and water soluble Si forms.

Consequently, the aim of our study was to identify absorption bands in FTIR spectra that are characteristic for the (i) amorphous silica (Sipernat, ZEOfree, Aerosil) as compared to quartz sand, silica gel, sodium silicate (air dried standard solution) and SROAS and (ii) water soluble fractions of amorphous silica compared to those of particulate silica. Our hypothesis were that (i) the Si–O–Si band in FTIR of amorphous silica fraction shows up at a region that is different to the ones of respective SROAS or sodium silicate and (ii) the FTIR of water soluble fraction of amorphous silica shows up bands at different WN as compared to those of the respective particulate silica. For the current study, we define the different analyzed compounds as follow: amorphous silica (amSiO_2_), the quadruple negatively charged and tetrahedral coordinated SiO_4_^4−^ anion of the monomeric silicic acid H_4_SiO_4_ (SiO_4_^4–^ orthosilicate) and the condensates of H_4_SiO_4_ as polymeric silicic acid, silanol the –Si–OH group within a silicon dioxide component. Silicates are salts of silicic acid and occur naturally as silicate minerals.

## Material and methods

The commercially available amorphous silica Sipernat 50, Aerosil 300, Sipernat 320, and Sipernat 50 s (all from Evonik Resource Efficiency GmbH, Wesseling, Germany), the calcium silicate ZEOFREE 600 (Evonik Operations GmbH, Wesseling, Germany) and sodium silicate (Na_4_SiO_4_) as well as two Andosol samples from Ecuador^23^ were analyzed by using Fourier Transform infrared spectroscopy in KBr-transmission technique (FTIR).

For each of the above named species about 0.5 mg sample was mixed with 100 mg of KBr (spectroscopy grade; Merck), stored overnight in an desiccator over silica-gel (to prevent water uptake), then finely-ground in an agate mortar and pressed into pellets^24^ that were finally analyzed by using a FTS135 (BioRad Corp, Hercules, CA, USA) (BioRad). All samples were analyzed in four replicates (n = 4). To obtain the FTIR spectrum of sodium silicate (Na_4_SiO_4_) about 1 mL of a sodium silicate solution (Merck Chemicals GmbH, Darmstadt, Germany) was placed at a zinc-selinid plate, and dried to obtain a white solid, and was analyzed using the FTS135 (n = 4).

Additionally, 10 g of Sipernate 50 s (Evonik Resource Efficiency GmbH, Wesseling, Germany) was mixed with 1 L of water, treated for 15 min within an ultrasonic bath (35 kHz; RK106; Bandelin Electronics, Germany), to ensure dispersion as far as possible. Thereafter, 100 mL aliquots of the mixture were transferred into 6 polyethylene cylinders (16 mm in diameter and 160 mm in height) (n = 6), stored for 48 h to allow precipitation of the Sipernat 50 s particles from the supernatant, and 10 ml aliquots were taken from the upper 50 mm of the supernatant. These aliquots were shock frozen by using liquid nitrogen. The shock-frozen samples were freeze dried and analyzed as described above by using FTIR.

Each FTIR spectrum was recorded by 16 co-added scans at a resolution of 1 cm^−1^ in the region of 400–4000 cm^−1^. All FTIR spectra were corrected against the atmosphere as background^25^, and smoothed using a „boxcar “-function (f = 105 for KBr-transmission technique and f = 25 for micro-FTIR; Win-IREZ software, BioRad). The spectra were baseline-corrected and normalized for the band at WN 1100 cm^−1^^24^.

The spectra were interpreted as follows: The bands at spectral regions between WN 3500–3300 cm^−1^ are characteristic for hydroxyl groups (O–H; stretching vibration) that are part of water molecule and silanol groups (Si–OH), the latter mostly located at the surface of the silica particles. The adsorption band at about 1630 cm^–1^ is attributed to the bending^19^ or deformation mode of molecular coordinated water adsorbed to the Si–O–Si structure (H_2_O band; Kaya et al.^21^, Roulia et al.^26^). While the bands at 1050–1100 cm^−1^ are the most significant spectral region (with regard to Si) and accompanying shoulders are attributed to asymmetric stretching vibrations of Si–O–Si. Lower internal order of the material under study may leads to peak broadening of the entire envelope^20^, denoted here as Si–O–Si band. Infrared spectra of SROAS showed an absorption band between 1018 and 975 cm^−1^ which shifted to lower WN with increasing Al:Si ratio and is attributed to stretching vibrations of the Si–O–Al bond (oxygen bridges formed by condensation of Si and Al hydroxides^19, 27^. The bands at wavenumber (WN) 980 cm^−1^ are characteristic for Si–O stretching vibrations of Si–O–H (Si–OH band) groups, which are attributed to asymmetric vibration of Si–OH. An increase of this bands is linked to an increase of OH-groups which are located at the surface of polymerized silica while those at WN 798 cm^−1^ are attributed to symmetric stretching vibrations of Si–O–Si^28^ and that at 467 cm^−1^ for Si–O–Si out of plane deformations. The intensity of the bands were each measured as a vertical distance from the maximum of the respective band to the baseline (as it was described for C–O–C bands in Kaiser et al.^29^). Though, Al–OH bending vibrations occur at WN 590–570 cm^−1^. A relative decrease of their intensities to the intensity of the Si–O–(Al) stretching region (WN 1018 and 975 cm^−1^) is caused by a decreasing Si content of the SROAS. Furthermore, Si-rich SROAS show an absorption maximum at 690 cm^−1^, while the bands at WN 430–440 cm^−1^ are related to vibrations of Si–OH groups^19^. Thus, these spectral regions are suitable to distinguish SROAS from “pure” Si phases. Though, the bands within the fingerprint region at WN < 1000 cm^−1^ in the FTIR spectra of the studied silica samples can be used to identify specific Si–O components.

## Results and discussion

**Figure 1:**
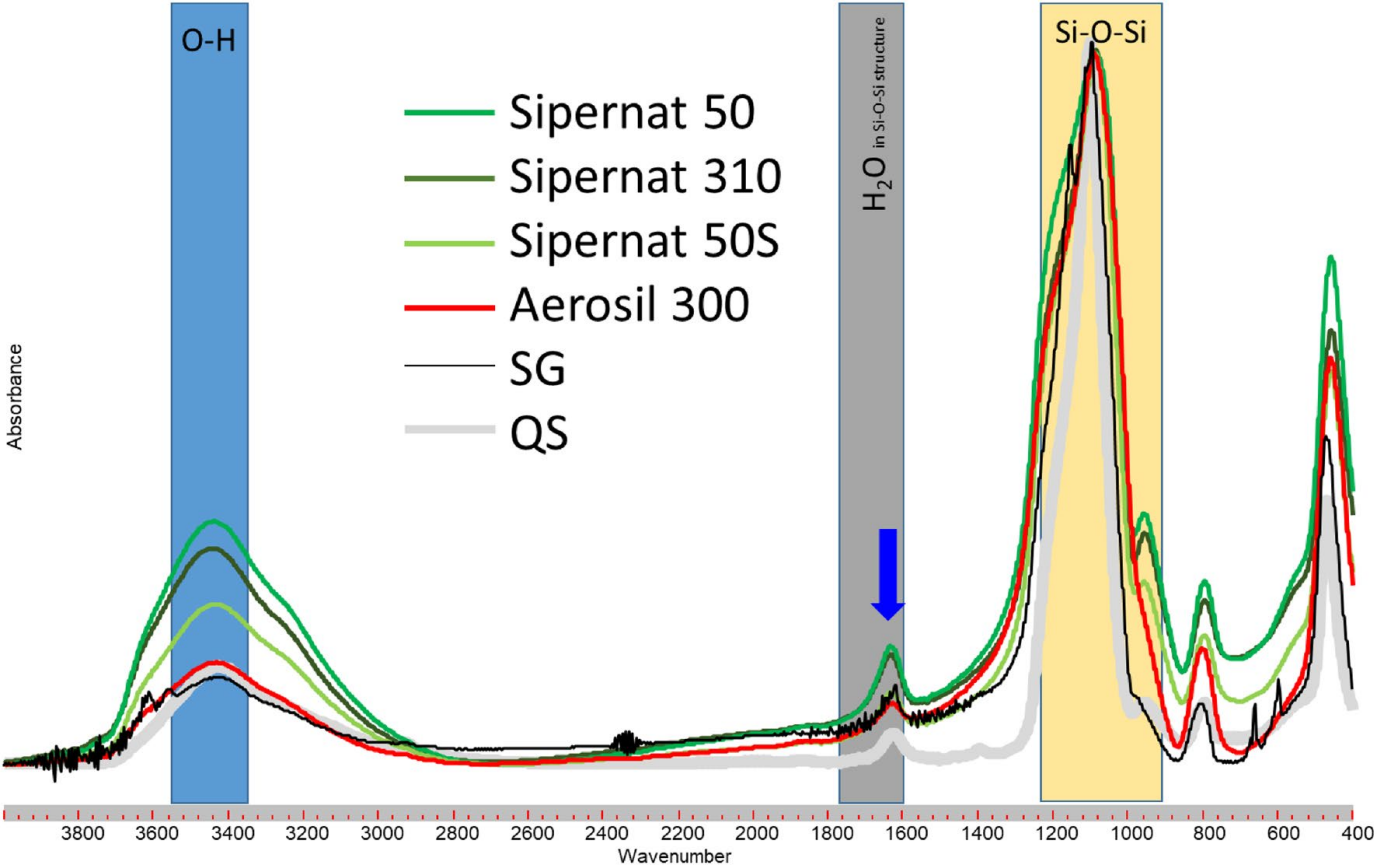
FTIR spectra of Sipernat 50, Sipernat 320, Sipernat 50S (each of them a precipitated silica; Evonik), ZEOfree 600 (ZEOfree, a precipitated Ca-silicate; Evonik), Aerosil 300 (Aerosil, a pyrogenic silica; Evonik), finely ground silica gel for chromatographic purposes (SG; Merck) and quartz sand < 63 µm (QS, Roth).

The FTIR spectra of all Sipernat 50 s samples (Sip) (Fig. 1) showed absorption bands characteristic for stretching vibrations of O–H groups (blue bar; OH band) at WN 3500–3300 cm^−1^, and Si–O–Si groups (yellow bar; Si–O–Si band) at WN of 1000–1100 cm^-1^. Additionally, the spectra show a band at WN 1600–1650 cm^−1^ (blue arrow) which indicate the presence of molecular coordinated water within the SiO_2_-structure^30^. The 1650 cm^−1^ band is less intense for Sip50s as compared to the other samples. A broadening of this envelope, an increase in intensity and/or peak shifts to higher WN may be attributed to a more complex structure of the material under study, which might result in a higher binding affinity towards water^19^. However, Sip50S showed a smaller particle size (18 µm) as compared to Sip 50 or Sip 320.

The FTIR spectra of Aerosil showed the lowest intensity of the O–H band, which is in accordance with the fact that Aerosil is a pyrogenic silica that may contain only small amount of water and OH-groups due to the conditions of synthesis. Pyrogenic or fumed silica is formed when silicon tetrachloride (SiCl_4_) reacts in a hydrogen flame (2100 K) to form amorphous silicon dioxide (SiO_2_)^31^. With this single spherical droplets of silicon dioxide form and is followed by particle growth through collision and coalescence forming larger droplets whereas the aggregation is occurring via cooling^31^.

As expected, the FTIR spectra of the Sipernat- and Aerosil-samples correspond mostly to that of silica gel (SG, Fig. 1: grey line). However, for the silica gel the Si–O–Si band is narrower as compared to that of Sipernat and Aerosil (Fig. 1, grey arrow). The spectra of Sipernat has of course more OH groups compared to the Aerosil due the formation process. The FTIR of quartz sand (QS, black line in Fig. 1) is also mostly comparable to that of the silica gel sample but shows a second maximum at the left-hand side of the Si–O–Si band (Fig. 1, grey arrow).

**Figure 2:**
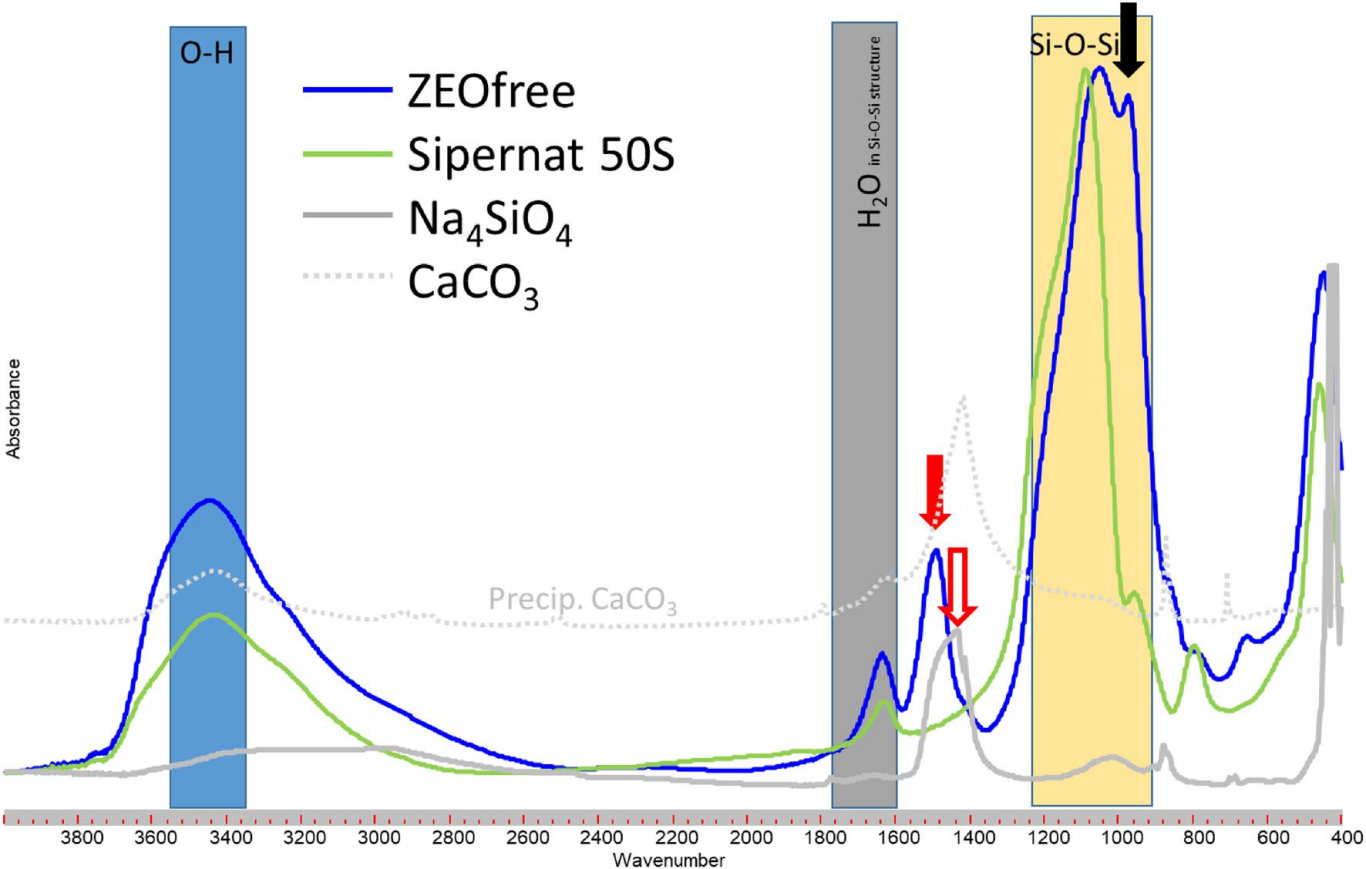
FTIR spectra of Sipernat 50 s (precipitated silica; Evonik), ZEOfree 600 (ZEOfree, precipitated calcium silicate, Evonik industries), calcium carbonate (grey dotted line) and sodium silicate (Na_4_SiO_4_) bold grey line to characterize monomeric SiO_4_^4−^).

The FTIR spectra of ZEOfree (Fig. 2), a precipitate calcium-silicate, showed an additional band at WN of about 1500 cm^−1^ (Fig. 2; red solid arrow) compared to the ones of Sipernat 50 and 320 (Fig. 1) and that of Sipernat50s (green line in Fig. 2), a shift of the Si–O–Si band towards smaller WN, and a second maximum at the right hand side of the Si–O–Si band (Fig. 2; black solid arrow). Molecular IR vibrations are a function of binding strength and the mass of the atoms involved. A band shift towards lower WN occurs as the mass of the atoms (here m > 28 u) increases. Furthermore, peak broadening may indicate a lower internal order of the material under study^18, 22^. The additional band at about 1500 cm^−1^ in the FTIR of ZEOfree (Fig. 2; red solid arrow) is in a similar range of WN as the SiO_4_^4−^ band of sodium silicate (Fig. 2, grey line, Fig. 2: empty red arrow).

**Figure 3:**
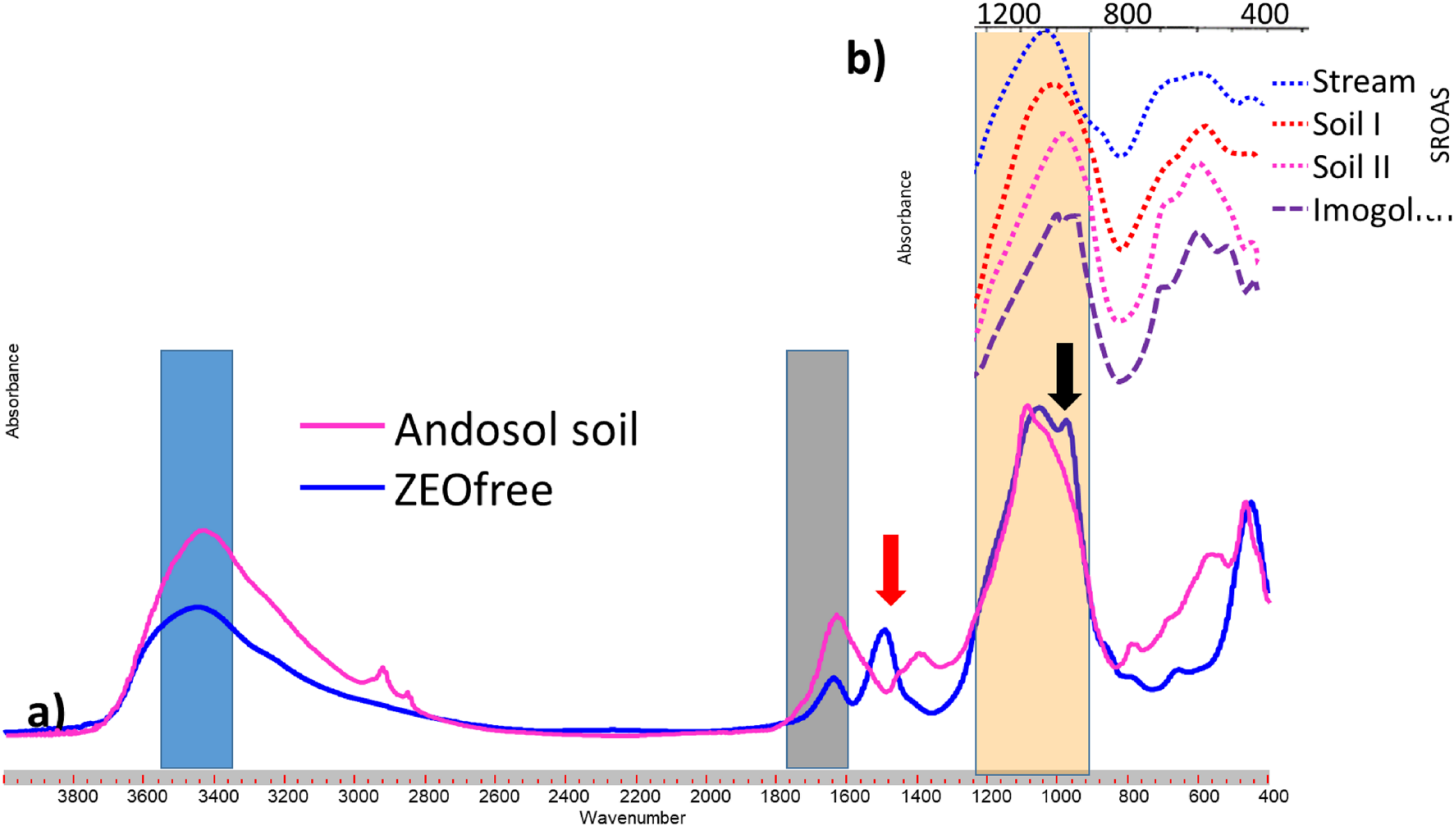
(**a**) FTIR spectra (400–4000 cm^−1^) of ZEOfree and an Andosol soil from Ecuador (Soil I + II) as well as (**b**) a 400–1400 cm^−1^ section of the FTIR spectra of SROAS, and imogolith (Imogol.; adapted from Parfitt^32^).

Compared to ZEOfree the FTIR spectra of the Andosol soils show no absorption band at about 1500 cm^−1^ (Fig. 3a, red arrow). The Si–O–Si bands of the Andosol soils look similar to those of the SROAS studied by Parfitt^28^ (Fig. 3b). However, the FTIR spectra of SROAS show a the shift of the Si–O–Si band towards lower WN (Fig. 3b; Parfitt^32^), which is found by Wada et al.^33^ to increase with Al content. However, there is currently some inconsistency in the definition of allophane. The definition of allophane commonly used in soils science from Parfitt^32^ stating that: ‘Allophane is the name of a group of clay-size minerals with short-range order which contain silica, alumina and water in chemical combination’ is misleading as allophanes are defined as particles consisting of spherical, hollow units^34, 35^. Short-range ordered silica, amorphous aluminosilicates and also amorphous silica are phases not belonging to the group of allophanes^4^. Only Imogolith (Fig. 3b, line A) showed a second maximum of the Si–O–Si band like ZEOfree a. The FTIR spectra of SROAS studied by Wada et al.^33^ (designated therein as allophanes) showed also absorption bands at WN 550–720 cm^−1^ (Fig. 3b) which are indicative for Si–O–Al bands. Most recently Lenhardt et al.^20^ reported on IR properties of SROAS with varying Al:Si ratios and identified SROAS specific absorption bands at 590–570 cm^−1^ (Al–OH bending vibration) which decreased with increasing Si content and an absorption maximum at 690 cm^−1^ for Si-rich SROAS. In contrast to the SROAS (Fig. 3b), the silica samples studied here (Fig. 1) did not show absorption bands at this WN.

**Figure 4:**
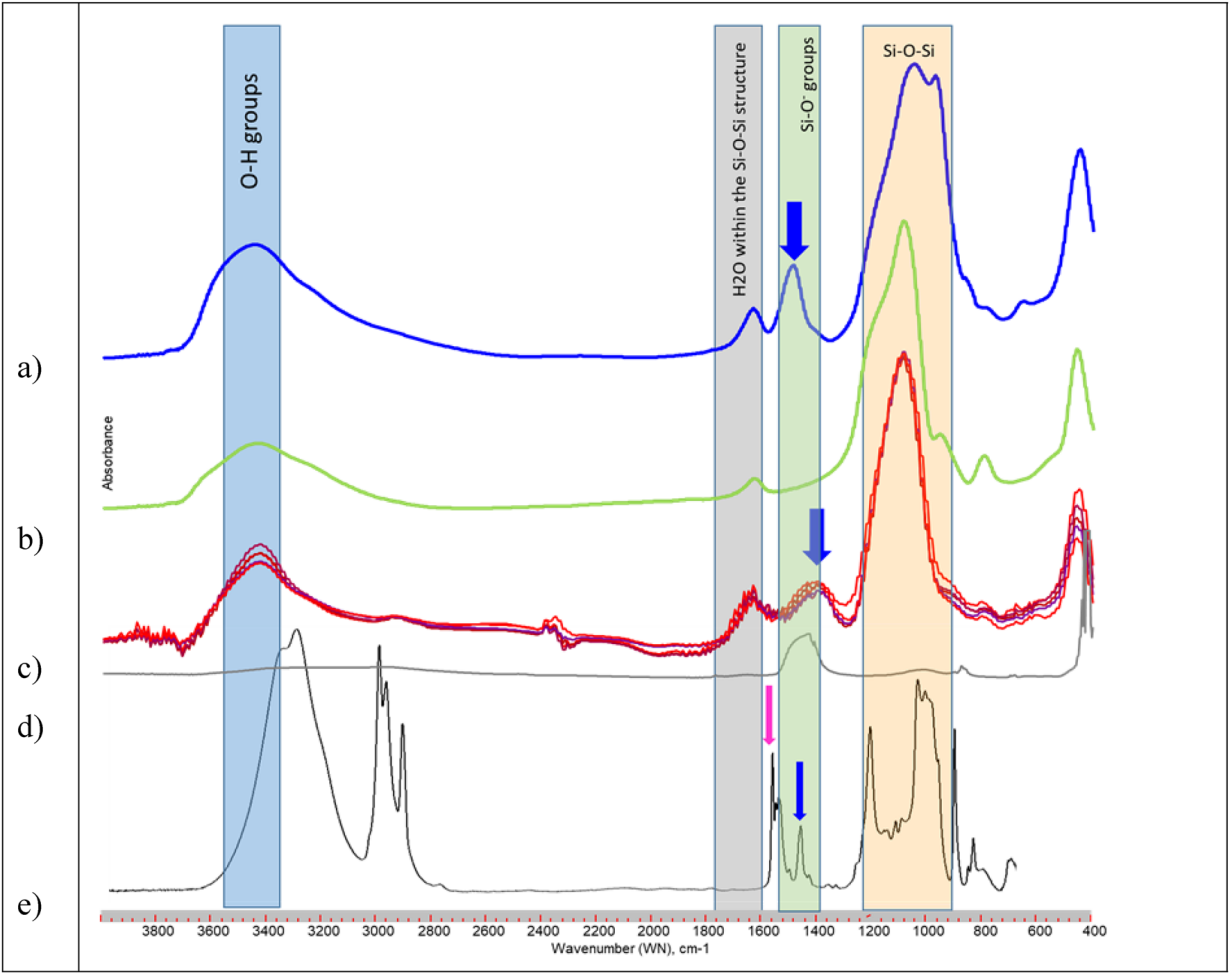
FTIR spectra of (**a**) ZEOfree, (**b**) bulk Sipernat 50 s, (**c**) water soluble Sipernat 50 s fraction (n = 6), (**d**) Na_4_SiO_4_, and (**e**) a dimeric organo-silica (adopted from Igarashi et al.^36^).

The FTIR spectra of the water-soluble fraction of Sipernat 50 s showed a smaller Si–O–Si band (ca. 1100 cm^−1^) and an additional band (Fig. 4c, bluearrow) at about 1400 cm^−1^ which is characteristic for Si–O^−^ groups compared to the bulk Sipernat 50S sample (Fig. 4b). The additional band in the spectra of the water soluble Sipernat50s fraction is located at a lower WN (WN 1400 cm^−1^) as compared to that of ZEOfree (1500 cm^−1^; Fig. 4a) and Na_4_SiO_4_ (1475 cm^−1^; Fig. 4d).

**Figure 5:**
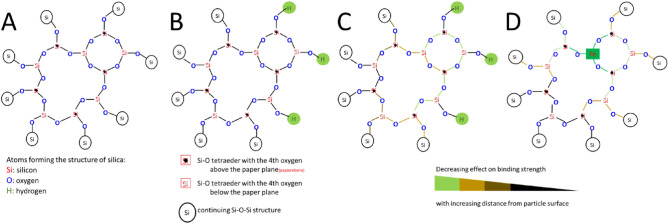
Schemes of Si–O–Si bonds (**A**) within the center of and (**B**) located at the surface of an amorphous silica particle. (**C**) scheme of Si–O–Si bonds near to particles surfaces with the different colors indicating the how binding strength of Si–O–Si groups is affected by the OH groups located at the particles surface and (**D**) a silica particle that contains an Fe cation including the cations effect on the binding strength.

The narrower Si–O–Si band in the spectrum of silica gel compared to Sipernat or Aerosil samples (Fig. 1, grey arrow) can be explained by differences in the specific surface area (SSA) of Sipernat and Aerosol samples (150–500m^2^ g^−1^) compared to the silica gel and quartz sand samples. With increasing specific surface area, the number of Si–OH increases and the number of Si–O–Si groups located near to the surface is increasing. Such the ratio between the number of Si–O–Si group located near to the surface (Fig. 5b) and that of the ones located in the particles center (Fig. 5a) will increase. This may be important since the presence of Si–OH groups is affecting the binding strength of neighbored Si–O–Si groups. This effect may decrease with increasing distance such that a closely neighbored Si–O–Si group will be affected more strongly (green colored bonds, Fig. 5c) than Si–O–Si groups that are located at larger distances (brownish to black colored bonds, Fig. 5c from the particles surface (i.e., within the center of a non-porous silica particle like silica gel). Since the number of Si–O–Si bond located near to the particles surface relative to the ones located within the particles center is increased with the specific surface area, the number of “affected” Si–O–Si groups increase with SSA. Such number of Si–O–Si groups different in binding strength increases with SSA that it may become detectable in the FTIR spectra of samples with higher SSA resulting in broader Si–O–Si bands for the Sipernat and Aerosil samples. Bands around 955 cm^−1^ may also act as an indicator for specific surface area (SSA) as they are attributed to the asymmetric vibration of Si–OH. An increase in intensity of this band indicated an increase in SSA as hydroxyl groups are mainly situated at the surfaces of the respective Si species^18^.

Sand grains (QS) with a low SSA also have Si–OH groups at the particles surface, which of course affect the binding strength of the neighbored Si–O–Si bond. But the low porosity and the relatively large particle size of such grains result a low SSA such that the number of Si–O–Si groups located near to the surface relative to the ones located within the particles center (Fig. 2) is much smaller for quartz sand as compared to amorphous silica like Sipernat. Consequently, mostly the Si–O–Si groups located within the particles center will show up in the FTIR spectra resulting in a small Si–O–Si band. Additionally, quartz has the highest internal order compared to the other materials analyzed which results in a narrow Si–O–Si band.

In the FTIR of quartz sand (black line in Fig. 1) the second maximum at the left-hand side of the Si–O–Si band (grey arrow, Fig. 1) can be explained by the fact that the quartz sand studied here is a commercially available material that is obtained by ashing and HCl treatment of sea sand. However, this procedure will not remove cations that are fixed within the Si–O–Si structure. Thus, the quartz sand will contain small amount of cations like iron within its Si–O–Si structure in contrast to silica (Fig. 5d): this cation substitutes a Si within the Si–O–Si structure. The presence of such cations within the Si–O–Si structure (Fig. 5d) will also affect the binding strength of the neighbored Si–O–Si groups and may explain the observed second maximum of the Si–O–Si band in FTIR (Fig. 1; black solid arrow) which may be interpreted instead as a Si–O–Fe band.

The band at about 1500 cm^−1^ (Fig. 2; red solid arrow) in the FTIR of ZEOfree may results from its high calcium content and its high pH value: both, the Ca content and the high pH suggest the presence of deprotonated -negatively charged- silanol (Si–O^−^) groups suggesting the formation of Si–O^–^–Ca^2+^ groups at the surface of the ZEOfree particles. Those Si–O^–^–Ca^2+^ groups will show Si–O^−^ bands in FTIR at a comparable but not identical WN range as the Si–O^−^ band of the sodium silicate (Na_4_SiO_4_). However, the Si–O^−^ band of ZEOfree showed up at WN 1500 cm^−1^ while that of the Na_4_SiO_4_ showed up at 1440 cm^−1^. The difference between the Si–O^−^ bands from sodium silicate and that of the ZEOfree samples can be explained by different chemical environment of the Si–O^−^ groups within the Na_4_SiO_4_ and the ZEOfree: in contrast to Na_4_SiO_4_ ZEOfree is not a monomer. Such Si–O^−^ group in ZEOfree is neighbored by Si–O–Si groups that will affect the binding strength of the Si–O^−^ band thereby causes a shift of the Si–O^−^ band in FTIR compared to that of Na_4_SiO_4_.

**Figure 6:**
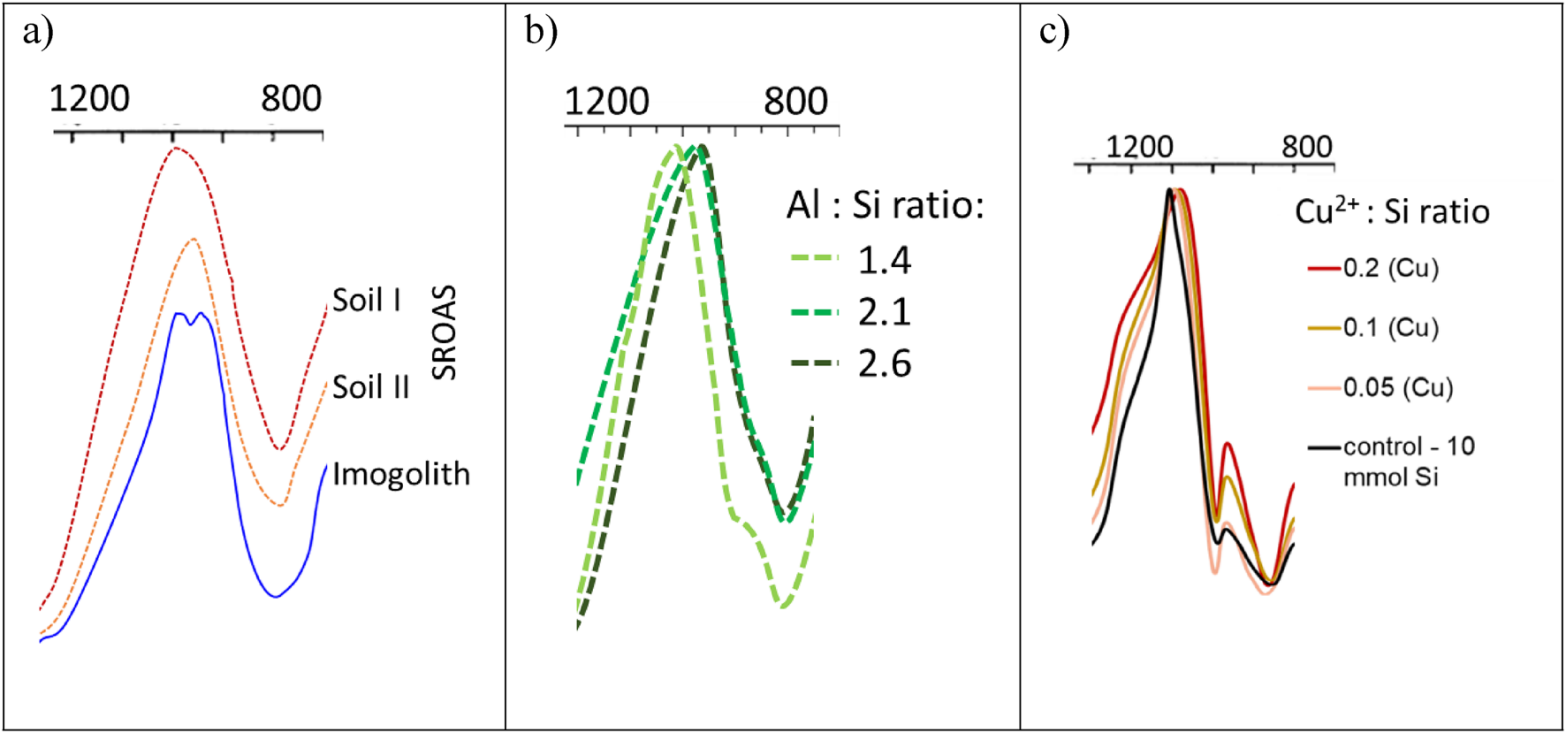
WN 800–1200 cm^−1^ sections in FTIR spectra of (**a**) soil allophanes and imogolith (adopted from Wada et al. 1979), (**b**) short-range ordered aluminosilicates SROAS with different Al:Si ratios (adopted from Lenhardt et al.^20^), and (**c**) polymerized silica associated with Cu^2+^ (adopted from Stein et al.^19^).

Like ZEOfree the Imogolith (Fig. 3b, line A) showed a Si–O–Si band with a second maximum. This may result from Al inserted into the Si–O–Si structure of SROAS which affects -like the iron traces in quartz sand (black line; Fig. 1; see above)- the binding strength of the surrounding Si–O–Si groups. Such Si–O–Al groups may explain the second maximum of the Si–O–Si bands. The SROAS showed also absorption bands at WN 550 and 720 cm^−1^ (Fig. 3b) indicative for Si–O–Al bands^32^, Wada et al.^33^ but, these bands did not did not show a second maximum for the Si–O–Si band (Figs. 3b and 3c). The same study found that increasing Al content increases a shift in the Si–O–Si band by comparing the FTIR spectra of SROAS with different Si:Al ratios (Fig. 6a). A similar shift was observed by Lenhardt et al.^20^ when analyzing short-ranged ordered aluminosilicates with varying Al:Si ratios (Fig. 6b). Stein et al.^19^observed a peak shift towards lower WN and a broadening of the entire envelope (1000–1300 cm^−1^) when analyzing silica metal compounds which were synthesized at undersaturation of silicates (Fig. 6). As the definition of allophane by Parfitt^32^ is wrong, we suggest that those compounds may be simple SROAS, amorphous aluminosilicas or ASi.

The FTIR spectra of the water soluble Sipernat 50 s fraction (Fig. 4d) showed an additional band that is in the WN range of the Si–O^−^ band found for ZEOfree (1500 cm^−1^; Fig. 4a) and Na_4_SiO_4_ (1475 cm^−1^; Fig. 4d) compared to that of the bulk Sipernat (Fig. 4b). This band indicated that the FTIR spectral signature of water soluble Sipernat 50 s-fractions can be distinguished from that of the bulk Sipernat 50 s, and suggests the water soluble fraction of Sipernat 50 s consist mostly of small sized amorphous, anionic silica particles, The pH values is about 6, such deprotonation of silanol groups may be neglectable small. The bands at 1400 and 1100 cm^−1^ in the FTIR of the water soluble SIP50s fraction possibly indicate the presence of dimeric silica, since they correspond to the respective ones in the FTIR of a dimeric organo-silica (adopted from Igarashi et al.^36^; Fig. 4e).

## Conclusion

As Si cycling, its speciation, and availability in soils is crucial to terrestrial ecosystem functioning and services the analysis of different Si species is highly important. Comparing amorphous silica, SROAS and silicic acid species using FTIR, we aimed to identify absorption bands that are characteristic for these different Si phases analyzed. Based on our results we report here that SROAS and Si phases containing traces of other elements in their Si–O–Si network can be distinguished from pure amorphous Si phases as their band positions and peak shapes of the respective IR spectra are specific for the bonds within the materials analyzed. Furthermore, liquid phases of silicic acid/water-soluble Si phases can be distinguished from the respective bulk material as water-soluble fractions showed an additional peak in its IR-spectra. As the current methods to analyze different Si phases are based on extraction procedures, which are not very selective^15^, we recommend using FTIR analyzes for such analysis. The limited number of synthetic samples and/or soil restricts generalization of our results regarding the estimation of differing Si phases in soil. Thus, further investigations using soils of varying properties with regard to their Si biogeochemistry are important to verify the described method and thus, overcome soil analyzes which are using unselective extraction agents to answer specific questions in terms of Si and its role for ecosystem functioning.

## Data Availability

The datasets used and/or analyzed during the current study is available from the corresponding author on reasonable request.
